# Organising housing and service provision for persons with co-occurring substance use and mental health problems: a scoping review in the ROP Municipal

**DOI:** 10.1186/s12888-025-07621-6

**Published:** 2025-12-11

**Authors:** Minna Sorsa, Unn Hammervold, Silje Lill Rimstad, Marianne Storm, Hildegunn Sagvaag

**Affiliations:** 1 Department of Public Health, University of Stavanger, Professor Olav Hanssens vei 10, Stavanger, Rogaland 4036 Norway; 2 Social Psychiatry (Public Health), Tampere University, Arvo Ylpön katu 34, Tampere, Pirkanmaa 33520 Finland; 3 Research Group of Nursing and Health Sciences, Research Department, Stavanger University Hospital, Stavanger, Norway; 4 Faculty of Health Sciences and Social Care, Molde University College, Molde, Norway

**Keywords:** Co-occurring disorders, Dual diagnosis, Housing first, Severe mental illness, Substance use disorder, Supported housing

## Abstract

**Background:**

Persons with co-occurring disorders, both substance use and mental health problems (COP) may be encountered within various disciplinary systems and organisational levels of treatment. In western countries, the most common evidence-based housing programme for persons with complex, long-term needs due to severe mental illness and substance use is Housing First. The context of this study were the Nordic countries, granting universal access to treatment and free public services. The aim was to examine how multidisciplinary and integrated housing services are organised and coordinated for persons with COP.

**Methods:**

We conducted a systematic search for literature in six databases (i.e. CINAHL, Ovid, SocINDEX, Web of Science, Scopus, and Social Services Abstracts), along with manual searches. After blinded review by two authors in Rayyan, the full texts of 75 articles were reviewed for inclusion, the quality of the selected research articles was completed according to checklists from the Joanna Briggs Institute. A thematic analysis of nine articles was completed according to a coding scheme.

**Results:**

From the Nordic perspective, introduced as a model in this article, regions need to develop the organisation and the workforce skills of health and social services as an integrated whole, with special focus on defining responsibilities at different levels, maintaining and improving relationships within a multidisciplinary, integrated, comprehensive, and community-based system of care inclusive of housing services. Supporting the recovery of persons with COP means giving them a voice and having professionals skilled in COP aid them.

**Conclusions:**

The approach of emphasizing primary care and multidisciplinary mental health is yet today a challenge even in high-income areas. The integration of services was not on an ideal level, even though the housing policies were rights-based. We identified societal responsibilities stemming from national policies. It is possible that functional integration does not take place in all regions. As the working methods were based on active relationship building to be able to help persons with COP, we interpreted this as a sign of clinical integration. The recommendations for the organisation and coordination of services for persons with COP include ensuring the right to a home, ensuring social integration and community involvement, combatting stigma, and systemising user involvement. There are research gaps in all Nordic countries and the research within housing for persons with COP is scarce. We call for using multi-dimensional research approaches.

**Clinical trial number:**

Not applicable.

**Supplementary information:**

The online version contains supplementary material available at 10.1186/s12888-025-07621-6.

## Background

In most Western countries, treatment for mental health conditions and problems with substance use continues to follow disparate treatment traditions [1]. Persons with both co-occurring substance use and mental health problems (COP) and thus complex needs may be encountered within various disciplinary systems and organisational levels of treatment [2, 3]. Although promoting mental health ranks among the United Nations’s Sustainable Development Goals and is of importance for societies as a whole [4, 5], major challenges remain in supporting persons with COP in different countries, regions, and areas, as well as with developing inclusive support for community mental health globally [4, 5].

In this article, we use the term *persons with co-occurring substance use and mental health problems with complex needs,* in order to discuss multimorbidity. By contrast, the usual biomedically oriented concepts of dual diagnosis, concurrent disorders, co-occurring disorders, and comorbidity may overlook aspects of the resilience and resources that individuals possess as they confront various challenges due to their multiple conditions. To promote compassionate care and welcoming approaches in treatment for persons with COP, increasing their engagement and following a whole-person approach are pivotal undertakings [3, 6, 7], not least because a fundamental understanding of human and individual change always needs to be person-oriented [6, 8].

Persons with COP have complex needs. The challenges and suffering experienced by persons with COP include diagnoses such as substance dependence disorders and Severe Mental Illness (SMI) (including schizophrenia, bipolar disorder, and major depressive disorder), all frequently requiring long-term care, and are at increased risk of suicide and drug-related deaths [1]. Persons with COP also endure a significant burden of physical illnesses, hence another major challenge for public health: persons with COP have a far lower life expectancy than the general population in the Western world [1, 9–11]. Individuals with COP are at increased risk of social marginalization and diagnostic overshadowing, where physical health symptoms are misattributed to mental illness. Such misinterpretations can lead to serious, even life-threatening, consequences [12].

Even though persons with COP often engage with services more than persons with either substance use or mental problems alone [13], their illness may also cause them to struggle to disclose their challenges to professionals [11, 14]. This difficulty is not solely a result of their conditions, but may also stem from structural and societal factors—such as stigma [1, 15], fear of losing access to treatment, financial support, or housing—which can discourage openness and trust. They are frequently described as ‘hard to reach’ and often carry the burden of traumatic life experiences, poverty, violence, social isolation, and systemic discrimination, including racism [16]. Meanwhile, from a medical standpoint, their problems may remain underdiagnosed and undertreated, and from a policy perspective, current clinical recommendations provide limited support or directions for structuring services and integrating care [1]. This is surprising, since co-occurring mental and substance use disorders were identified as early as the 1980s [17]. Yet, the complexity is even more challenging, since many persons with COP have histories with imprisonment [1, 13].

The heterogeneity of persons with COP in their complex situations has caused challenges and needs with developing adequate resources and support services for many organisations and providers in systems of care [15, 18]. In questions about housing, for instance, inclusiveness and lowering barriers to owning homes has meant implementing flexibility, harm reduction, and motivational interviewing strategies to promote health [7, 8]. Persons with COP indeed often have poor housing circumstances or suffer from homelessness [5, 15] and therefore seek out many types of housing services. The *right to adequate housing*, internationally recognised in the UN Universal Declaration of Human Rights (1948), is defined as the right to live somewhere with security, peace, and dignity [19]. Secure housing is also among the Sustainable Development Goals, which state that communities should guarantee a home for everyone [5]. One model based on the right to adequate housing is Housing First (HF), a harm-reduction model suitable for persons with COP developed in 2004 [20] that has been contrasted with the Treatment First model, providing housing without requiring the person to attend treatment or demonstrate sobriety [21]. Therein, in line with a recovery-oriented framework, the service user occupies the role of tenant or citizen, whereas housing providers assume the role of facilitators [22]. The HF model, especially in combination with Assertive Community Treatment (ACT) teams, is preferred by service users due to its person-centred and flexible approaches [23], it has shown effectiveness in improving housing stability [7], and it helps to prevent the service gaps for people with SMI (including persons with COP). ACT is an active outreach treatment approach which delivers comprehensive, multidisciplinary support to adults with severe mental health problems—particularly those who are disengaged from traditional mental health services and persons with COP. The model emphasizes assertive engagement, continuity of care, and a high level of service intensity [24, 25].

Collaboration, mutuality, empowerment, having a voice, choice, and individuality, along with building safety and peer support within cultural, historical, and gender-sensitive contexts, have been recommended in trauma-informed approaches [3, 7, 16] alongside harm reduction strategies. There is a contradiction between the right to housing independent of changes in behavior and the fact that the right to housing requires simultaneous treatment. The HF model as an example, ideally includes practices of increased client choice side by side with professional harm-reduction strategies [7, 8]. According to Watson et al. (2013) misinterpretations can lead to the adoption of abstinence-based requirements of change in behaviour, which conflict with harm-reduction principles and undermine the model’s core values and thus also human rights [8]. Such deviations may place service users in a vulnerable position, where maintaining housing becomes contingent on compliance with conditions that contradict the right to housing independent of changes in behaviour, as the original intent of HF example—namely, unconditional housing and support [8]. Because recovery occurs within the context of everyday life—encompassing the evolving sense of self, social relationships, and living conditions [6, 26],—health as a human right must be addressed through multidimensional approaches. These approaches in the community require sustained investments in societal domains that foster both individual and collective mental health [5, 27, 28]. Accordingly, the social context of recovery includes relational, cultural, material, and societal dimensions, all of which interact with the lived conditions of daily life [6, 29]. In this article, we want to lift the perspective on housing and coexisting problems to a wider societal perspective including communities. Taking a recovery-oriented health promotion approach in organising services for persons with COP at the community level may be increasingly relevant as promoting health ideally becomes increasingly inclusive across sectors and supports people with taking control of their personal health [4–6, 27]. The recovery orientation encompasses areas of mental health that persons with COP may feel that they have lost, including a strong identity and a sense of meaning in life, of empowerment, of being connected, and of hope for the future [30].

The multifaceted nature of COP also impacts and creates challenges in organisation and delivery of coordinated care and treatment. According to Minkoff and Covell (2022) the integration of mental health and substance services is underdeveloped. However, components of systems and models of service use have proven to work well for persons with COP, including policies of inclusive communities and involving consumers as participants [2, 3], a recovery orientation [17], the development of regulations and legal processes [2], financial arrangements [2], information system capacities [2], workforce competency and training [2, 3, 17], coordinated treatment [1, 3, 11], the implementation of collaborative care [1, 3, 13], the integration of treatment [13, 17, 31], and increased collaboration across systems of care (e.g. justice, primary health, housing, social services, and child protection services) [3, 11, 14, 17, 31]. Helping persons with COP in a comprehensive and holistic way requires incentives from organisations for developing capable housing services with one or several providers in specific areas [2, 3, 17]. A challenge may be the lack of integrated services at the systemic level, ensuring that providers have sufficient skills in supporting persons with COP [17, 32]. Integrated care is an approach to overcome care fragmentation [32–34]. Today’s health systems policies require integrated support for persons with COP [1–3, 17, 35, 36]. From a practical perspective, integrating a care model that promotes mental health involves welcoming services, a person-centred, equity-based approach [3, 15, 17]. Such integration of programmes and interventions may occur between several levels, including the ethical level, the systemic level, and the level of service organisation [17, 37], and in such cases, the different levels of integration and types of services integrated require the development of skills and attention from the system’s leaders [3, 17]. To summarise, integration has been completed at clinical level (micro), in professional level (meso), in organizational level (meso), and also within a functional (macro) or normative (macro) integration of all levels [33].

Primary care plays a critical role in mental health conditions through screening and in delivering verbal, psychosocial, and pharmacological interventions [4, 11]. Nevertheless, significant barriers remain in effectively implementing strategies for identifying severe mental illnesses within primary care services [4, 11]. However, primary care providers often lack the specialized competencies required to address the complex needs of individuals with COP, thereby creating barriers to effective support [24, 38]. On the other hand, primary care can also be less stigmatising than mental healthcare [10]. Research and best practices have not been implemented into primary care in all western countries [9, 24]. Guidelines for clinical management have also been developed for COP [1, 3, 13], but more research on strategies to implement the integration across systems remains necessary [3, 7, 17]. There are also unidentified valuable resources outside official social and healthcare services, including user organisations and churches delivering food assistance, contributing resources, especially for promoting recovery and health.

### International research, research gap and recommendations in the Nordic context

Internationally, research on guidelines for practice and for implementing services for persons with COP in the field of housing remains scarce. One exception is the work of Alsuhaibani et al. (2021) [35], who identified three guidelines for persons with COP within a comprehensive approach. In all these, a risk of homelessness for persons with COP was mentioned, and two of these discussed housing assistance. There is a lack of integrated knowledge on the organization and networking of housing and service provision for COP.

Differences in service organisation may arise depending on the setting in which services are administered. Some areas may have developed services within a research framework focused on their own region—for instance, Canadian provinces [13]. Our perspective in this article is the Nordic countries—Denmark, Finland, Iceland, Norway, and Sweden—where the development of health and social policies has occurred at national, regional, and local levels in the fields of mental health and social service, as well as in housing policies, to help persons with COP holistically, e.g by incorporating a flexible ACT to help prevent the gaps for persons with COP. In the Nordic context, service provision has increasingly shifted toward primary care [17].

In general, the Nordic welfare societies have provided their citizens services within a framework of equality and universal access to care, and context of their societies and systems is based on universalism and the strong role of government. All citizens are included in a welfare state, and a comprehensive public sector is responsible for tasks of basic welfare, including social security and services. The welfare model, financed by taxes, aims to meet citizens’ basic needs for social care, healthcare, and education, among other things. However, challenges have included that the services are often viewed as fragmented, and access to proper treatment and housing for persons with COP does not always function. The Nordic countries also largely base their guidelines on substance use and mental health on the principles of evidence-based practice (EBP). EBP involves the use of the best available research, clinical expertise, and patients’ values and preferences to make decisions about health services [4].

Although Nordic welfare societies are similar, their drug policies differ, affecting views on criminalization, punishment, and health treatment for drug use for persons with COP. Bjerge et al. (2016) found that drug users are viewed as ‘alien’ in Finland and Sweden, while in Denmark, drug use is seen as a social problem [36]. Their opinion is that Denmark addresses drug problems with voluntary treatment based on meaningfulness, choice, and responsibility. In contrast, Sweden and Finland adopt a more moralistic approach, with Sweden being paternalistic and conservative, followed by Finland. Bjerge et al. (2016) do not compare Norway, where drug policy has shifted from being seen as a moral or a social problem to a societal issue. In 2004, Norway defined persons with drug problems as ‘patients’ and placed treatment responsibility on interdisciplinary specialized health services [37].

Cultural values, specific welfare systems, policy frameworks, and geographical considerations unique to the Nordic countries may have impacted organising services for persons with COP. Therefore, in our study, we carried out a scoping review to map the research literature to examine how housing services are organised and coordinated in the Nordic context. We specifically aimed to:

- Identify research gaps in the areas of organisation, coordination, and networking of housing and service provision for people with COP in the Nordic context; and
- Explore research-based recommendations for housing and service provision for people with COP in the Nordic context.

Throughout our research, we maintained a multidisciplinary perspective, by being open in the search terms for diverse professional fields on integration while remaining open to different research perspectives on the topic.

The review has been conducted in ROP- MUNICIPAL-Project, which is a collaborative research project between a medium-sized Norwegian municipality and University of Stavanger. The project aims to monitor the establishment and operation of a housing and service development for individuals with co-occurring substance use and mental health problems (COP). The primary objective is to develop new approaches to engaging with persons with COP and to identify legal and organisational barriers that hinder the provision of necessary and adequate support. The project explores how individually tailored housing and services for persons with COP and severe violence-related challenges, can be developed in alignment with the needs of persons, their relatives, and professionals—within the framework and structure of the existing Norwegian welfare system.

## Methodology

Scoping reviews are suitable for summarising literature on a topic in order to identify gaps in research in the domain and to make recommendations that inform practice in the field. Scoping reviews can address questions of the appropriateness, meaningfulness, and/or feasibility of healthcare practices. To access such information, systematic reviews need to target literature that is relevant to the context [39]. To answer our clinically meaningful questions, we decided to appraise the quality of the selected research articles in order to enhance the utility of the results for practice [40]. In reporting the results, we follow the PRISMA guidelines Extension for Scoping Reviews (PRISMA-ScR) [41].

### Search strategy

**Fig. 1:**
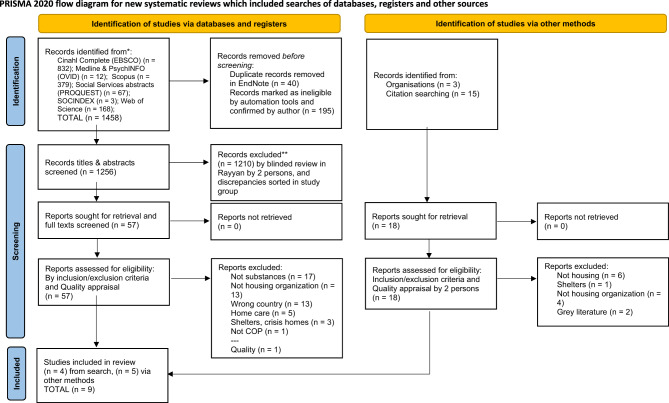
Flow chart of the literature search and selection process

Our search for literature was conducted on 15 February 2023 by the first author with the assistance of an information specialist in the following databases: CINAHL, Ovid (i.e. Medline and PsycINFO), SocINDEX, Web of Science, Scopus, and PROQUEST's Social Services Abstracts. We additionally searched the references of the included studies and remained open to any relevant literature found online during the process. We followed the PRISMA statement for reporting systematic reviews [42], as shown in Fig. 1. Our search through the titles, abstracts, and keywords of literature using a Boolean operator combined concepts related to housing, complex life situations involving severe mental illness and substance use, and an array of concepts related to the organisation of services, including at the level of praxis. Our search is detailed in Supplementary File 1, and all searches are available from the first author upon request.

### Inclusion criteria

The full inclusion and exclusion criteria for our review appear in Table 1. Only research articles written in English or a Scandinavian language were eligible for our review. Within that range of literature, we focused on any type of housing, including supported housing, residential treatment (e.g. HF), and single flats by any provider or within any system of organised housing. Housing means different housing models and homes where people live and pay rent. In some cases, the differentiation of institutions is very clear; however, institutions, crisis shelters, and crisis homes were excluded, along with housing facilities for older adults such as nursing homes, because our target group was 18–70-year-olds suffering from COP. We also excluded articles on homelessness and COP unless they focused on housing. In this article, we define COP as requiring long-term service use for a person diagnosed with SMI, including schizophrenia, psychosis, bipolar disorder, and major depressive disorder [15], in combination with a substance disorder, problematic substance use, or an ongoing substance problem. We did not consider a history of violent behaviour as an inclusion or exclusion criterion. Following the strategic guideline explicating that a thorough list of diagnoses is unnecessary, we assumed that persons with COP can show resilience and that tenants with COP have complex needs. In turn, because support for such individuals is organised and coordinated across sectors, we also included articles addressing tenants with COP and community integration, assertive outreach, neighbourhoods, and/or studies with information on funding and costs. The services could be provided by the public sector or private services and user organisations.

**Table 1:** Inclusion and exclusion criteria

Inclusion	Exclusion
Any type of housing, including supported housing, residential treatment such as housing first, single flats by any provider or within any system of organizing housing.	Institutions, crisis shelters and crisis homes
18–70 year old adults suffering from COP	Elderly housing facilities such as nursing homes
Peer-reviewed research articles	Homelessness and COP, if there was not a focus on housing
Research from Nordic countries (Denmark, Finland, Iceland, Norway or Sweden)	Other types of publications
Written in English or any Scandinavian language	Severe mental illness studied without substance use
	Severe substance use studied without severe mental illness

The choice of references from the Nordic countries from high-resource settings was made due to local contextual political and cultural features that differed from features in other Western countries. No such contextual review on the Nordic context has ever been conducted in the research domain. We found a vast amount of references on housing and mental health conditions, yet it was the stage of full-text review that allowed us to identify whether severe substance use or substances were included in the studied populations. It seems that most research in the area has not included persons with COP, both substance use and mental health problems at the same time. Therefore, the included references did not include articles from Denmark and Finland, even though we identified many references from those countries in our initial search. It seems that most housing study projects in the Nordic context have been completed for persons with SMI but not persons with COP (i.e. by excluding co-occurring substance use).

Our inductive grass-roots approach means that we refined and elaborated the analysis by building an inductive coding scheme (Supplementary material 2). We had to conduct several stages of working definitions [32]. For instance, during the selection of articles, the division between institutions and living in safe, supportive housing facilities was not especially clear, because there are so many different types of housing. There were also differences in whether the organisations would provide housing only or also treatment, care and assisting in integration with the communities, as research from Nordic countries has shown [41–43].

### Process of selecting studies

Our initial search yielded 1458 citations. All citations were blinded in Rayyan software and screened for inclusion by having two authors examine their titles and abstracts. In case of discrepancies, all authors discussed the prospect of inclusion and made a collective decision. We identified another 18 citations via organisations and reference lists search, and a second round of review was completed following the same steps as the first, and we ultimately included nine articles for a final, thorough analysis. The PRISMA flowchart of the inclusion process appears in Fig. 1 [44].

The quality of the articles was appraised independently by two authors using critical appraisal tools of the Joanna Briggs Institute, specifically to gauge the methodology of each study [43], as shown in Table 2. Discrepancies that arose were discussed among all authors, and all authors evaluated the overall quality of the articles by scoring them according to mentioned quality appraisal tools. Articles that received scores less than 75% were excluded from analysis, and one article was excluded due to low quality.

### Analysis

In the first step of analysis, the first author developed an initial inductive coding scheme by reviewing the articles. The way of developing such a conceptual framework has been recommended for complex interventions [45]. The coding scheme was reviewed by all authors and refined after a preliminary read of Nordic policy guidelines and recommendations for the field [46]; the final analytical framework is available in Supplementary File 2. Next, two authors piloted the framework to analyse the nine articles from the Nordic countries. Data extraction from the articles was completed primarily by the first author into predefined data extraction sheets, and two articles were subjected to double-screening (22% of the total) to evaluate the clarity of the questions. Most data were directly retrieved from the articles via the open-ended questions, some questions required interpretation to be answered. In the stage of reporting the results, we simplified the results into the tables. We were careful to extract and present the data in a structured way [40] and in relation to the questions guiding our review [41]. The ROP Municipal project also included a document analysis of Nordic governmental documents and strategic guidelines relevant to persons with COP that involved extracting all text describing the organisation of housing services and additional service provision, best practice guidelines, and recommendations in the strategic guidelines. That analysis, published in a separate article, afforded insights into the questions about context [46].

## Results

In what follows, we report the results on the organisation, coordination and networking of housing services for persons with COP from a Nordic perspective. The articles reviewed (Table 2) were all from two Nordic countries: five from Norway and four from Sweden. The articles were published from 2006 to 2022, with most written since 2018; in fact, only one was older (i.e. from 2006). Two of the studies in the articles followed a cross-sectional design, and seven were qualitative in nature. Two studies used the same original data.

**Table 2:** Background information of the included studies, and JBI Quality Appraisal

Authors	Year	Study context and population	Language	Title	Aims/purpose	Data	JBI Critical Appraisal tool
Blid, et al.	2006	Sweden	English	Socially excluding housing support to homeless substance misusers: two Swedish case studies of special category housing	To present findings from two case studies (settings) of special category housing for former homeless substance misusers.	Interviews with 26 staff members and 19 residents. Quality of life rating by 19 residents. Administrative and register data on residents during 10 years.	Qualitative Research, Scoring 85%
Hansen, I	2018	Norway	English	Users’ Choice in Providing Services to the Most Vulnerable Homeless People	To discuss the users’ experiences from receiving social support as part of the Housing First Programme.	Interviews with 16 participants in two Housing First trials in 2 municipalities.	Qualitative Research, Scoring 80%
Lydahl & Lofstrand	2020	Sweden	English	Doing good: autonomy in the margins of welfare	To inquire into modes of doing good care during professional workers’ home visits by building on observations of service interactions taking place during these home visits in two different settings.	Field notes and audio-recorded interactions from 16 home visits in special-housing unites and 15 home visits in the mental healthcare unit.	Qualitative Research, Scoring 80%
Matscheck, et al.	2019	Sweden	English	The Coordinated Individual Plan - is this a solution for complex organizations to handle complex needs?	To explore collaboration as it is indicated in Coordinated plans and other case documentation with focus on how the plans are motivated, and what kind and degree of collaboration is indicated by the documentation.	Examination of 12 individual case files.	Qualitative Research, Scoring 80%
Nesse, et al.	2020	Norway	English	Recovery, quality of life and issues in supported housing among residents with co-occurring problems: a cross-sectional study	To explore residents’ self-reported recovery and quality of life and examine the relationships between these factors and issues in supported housing.	104 residents from 21 supported housing sites responded to measures of recovery (Recovery Assessment Scale), life satisfaction (Manchester Short Assessment of Quality of Life), affect (single items), staff support (Brief INSPIRE) and sense of Home (single items)	Analytical Cross-sectional, Scoring 88%
Nesse, et al.	2022	Norway	English	The role of occupational meaningfulness and citizenship as mediators between occupational status and recovery: a cross-sectional study among residents with co-occurring problems	To examine associations between occupational status, occupational meaningfulness, citizenship and recovery and quality of life and to examine the roles of occupational meaningfulness and citizenship as possible mediators between occupational status and recovery and quality of life.	104 residents from 21 supported housing sites responded to measures of recovery (Recovery Assessment Scale), life satisfaction (Manchester Short Assessment of Quality of Life), Occupational status, source of income and prior housing situation, and citizenship (Citizenship Measure)	Analytical Cross-sectional, Scoring 81%
Nordaunet & Andvig	2018	Norway	Norwegian	Møte mellom skadereduksjon og Housing First i Norge -Ansattes beskrivelser	To evaluate a municipal Housing First (HF) program offering assertive community treatment with the principles of harm reduction.	5 focus groups with 5 workers.	Qualitative Research, Scoring 75%
Ogundipe, et al.	2022	Norway	English	Social recovery and economy: a thematic analysis of staffs' experiences with promoting social community participation for residents with co-occurring problems	To explore and describe staff working in a Norwegian supportive housing site’s experiences with promoting social community participation for residents with co-occurring problems.	9 staff members in qualitative interviews.	Qualitative Research, Scoring 75%
Von Greiff, et al.	2020	Sweden	English	Supporting recovery in social owrk with persons having co-occurring problems -clients’ and professionals’ perceptions	To identify recovery supportive components in treatment of co-occurring mental health and alcohol or drug problems.	40 client interviews and 15 interviews with professionals.	Qualitative Research, Scoring 80%

All articles reviewed included tenants with COP (Table 3). To describe the users in their corresponding studies, none of the articles use the term “patients”; instead, five of the nine articles use “persons”, two use “users”, two others use “tenants” and/or “residents”, and the last uses “citizens” and “clients”. The definitions of *mental ill health* varied such that six articles refer to severe illnesses, five refer to mental health problems, and two refer to disorders. The substance-related definitions refer to substance abuse problems in four of nine articles, to severe disorder in four other articles, and problems with alcohol and drugs in one article. COP are related to complex consequences and a long-term perspective in nine articles, and six articles use the term “comorbidity” or “co-occurring problem”. Only one article uses the term “dual diagnosis”, two discuss the long history of using different welfare services, three discuss complex needs or problems, two refer to “marginality”, and one mentions a high level of need for support, extensive support, how persons with COP require many kinds of help, highly diverse problems, and the social isolation they endure.

**Table 3:** Contextual information of the included studies

Authors	Year	Definition of the target group	Mental ill health definitions	Substance use definition	Complex consequences	Housing or housing + treatment	Housing model	Safety and security in housing facility
Blid, et al.	2006	People	Severe mental disorder	Severe problems of substance misuse	Psychiatric comorbidity	Housing without treatment	Flats owned by municipalities	Front door locked during the night, no violence or threat were allowed against other tenants or staff. Formal agreements.
Hansen, I	2018	Person	Severe mental illness	Substance abuse problems	Long history of contact with different welfare services, complex needs, high support needs	Housing and support from staff	Flats in different areas according to the wishes of the tenants	Establishing a safety plan together with staff on a very practical level including preferences for treatment in different situations.
Lydahl & Lofstrand	2020	People	Severe mental illness	Substance abuse problems	Complex needs, margin of welfare	Housing and support to own home	Special housing unit	Generally risks of rule breaking or bad behaviour
Matscheck, et al.	2019	Persons, users, tenants	Mental health problems, severe mental health disorders	Substance abuse problems, substance use disorders	Complex problems, Co-occuring, requiring many kinds of help concurrently	Housing and support from wide service network	Social housing, flats hired by tenants, housing with family	
Nesse, et al.	2020	Persons	Severe mental health problems	Substance use	Residents who experience problems, co-occurring problems are highly diverse	Housing and support from staff	Flats hired by tenants	Personal safety was connected with the possibility to receive support from staff, and satisfaction with the neighbourhood, the need to create a sense of a home
Nesse, et al.	2022	Citizens	Mental illness	Substance abuse	Co-occurring problems, dual diagnosis	Housing and support from staff	Flats hired by tenants	
Nordaunet & Andvig	2018	Users, persons	Mild to severe mental health problems	All types of substance problems	Harm reduction to people to handle their substance issues and mental ill health	Housing and assertive community treatment (support from staff)	Flats in different areas according to the wishes of the tenants	Keeping flat in spite of substance use: basic security. Developing crisis plans.
Ogundipe, et al.	2022	Persons, residents	Mental health problems	Substance abuse problems	Social exclusion, adverse living conditions, experiencing co-occuring problems and social isolation	Housing and staff available (housing solely is insufficient)	Housing unit	
Von Greiff, et al.	2020	Clients	Problems with mental health	Problems with alcohol and drugs	Long and frequent contact with services, marginalization, extensive support, co-occuring problems	Housing and support from wide service network	Supported housing	Physical milieus as a whole, safety is produced collectively

In our review, we focused on long-term illnesses and/or the use of services in all nine articles, all of which reported studies on questions of housing. Although no study focused solely on homelessness, some studies addressed the housing of homeless individuals as a starting point. Only one study investigated housing without other mechanisms of support, and in eight articles, the persons with COP were given various degrees of support from housing services or the larger community and specialised services (Table 3). In most studies, the persons with COP had rented flats, and four of the nine articles mention a specific housing unit (Table 3). The articles generally mention the complexity and variety of the type of problems faced by persons with COP. The physical milieu of the home was considered to be a whole where the feeling of safety was co-produced with other tenants as well as providers [47]—for instance, by establishing plans for safety or formal agreements [48, 49] that were supported by providers [50].

**Fig. 2:**
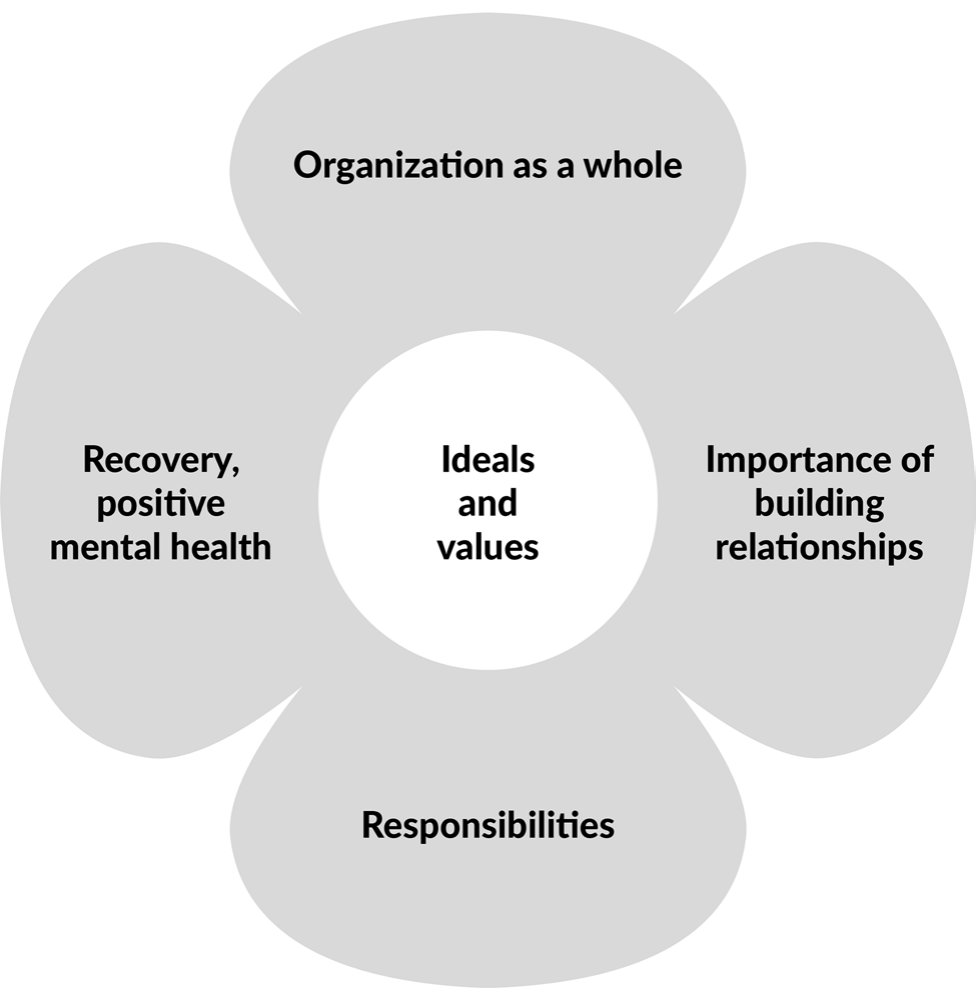
The model for persons with COP from a Nordic perspective

The results of our review using inductively formed themes are next presented. The themes are organisation as a whole, societal responsibilities, ideals and values, active relationship building, and recovery, positive mental health (Fig. 2).

### Organisation as a whole

Table 4 describes the organisation of services for persons with COP following a multidisciplinary approach and in terms of service integration, the accessibility and availability of services, skills development in the system of care, continuous evaluation, and an interest in measuring the meaningfulness of tenants’ lives according to quality of life measures.

**Table 4:** Organisation as a whole

Authors	Year	Multidisciplinary approach and service integration	Accessibility and availability	Skills development in systems of care	Continuous evaluation
Blid, et al.	2006	No	No	Staff wants training to benefit COP	Yes: housing stability, substance use, contact with services, safety in home, quality of life
Hansen, I	2018	Yes	Yes	Education in joint reflective practices	Yes
Lydahl & Lofstrand	2020	Yes	Yes	Staff performance is key to success	No
Matscheck, et al.	2019	Yes	No	Local collaboration	No
Nesse, et al.	2020	No	No	Recovery orientation	No
Nesse, et al.	2022	Yes	No	Meaningfulness and citizenship	No
Nordaunet & Andvig	2018	Yes	Yes	The attitudes of staff	No
Ogundipe, et al.	2022	No	No	Community participation	No
Von Greiff, et al.	2020	Yes	Yes	Flexibilty, skills in COP therapeutic alliances, secure physical milieus	No

Altogether, six of nine studies in the articles followed a multidisciplinary approach involving integrated services. In housing, service providers were shown to come from highly diverse educational backgrounds, with specific experiences limiting the content of their work and assignments [49]. Although multidisciplinary providers were found to have expertise in managing housing, they may have wanted to be trained in additional areas of competency suitable for persons with COP, including motivational interviewing, relapse prevention, care management, and providing advice [49]. Generally, “the home turn” meant that services would be administered close to the users’ homes [51]. The professionals mentioned specialised in housing, case management, mental health conditions, or substance use, and each professional had an area of expertise as physicians, nurses, psychologists, social workers, or physiotherapists. There was both the formal and informal use of collaboration [52], and they could function at different levels of practice, including in primary care, in employment agencies, in criminal justice services, in ambulatory services, and in specialised services such as psychiatric care systems or substance-related services. Because persons with COP have many problems that may vary in severity over time, the amount of support and providers needed in different periods varies as well [52]. Assertive community treatment was also mentioned as an integrated model [53].

Questions about the accessibility and availability of suitable housing services were addressed briefly in four of nine studies. One found that a long-term perspective for the organisation and availability of housing is ideal [48]; another found that providers were creating the supportive atmosphere in the housing facility [47]; and two showed that the around-the-clock availability of providers is supportive [47, 51]. Meanwhile, Nordaunet and Andvig (2018) [53] have discussed the location of housing within municipalities [53]. However, our questions related to access to housing are not discussed in the articles (i.e. whether flats are available, the time to queue, and how long persons with COP can stay in specific homes).

The development of provider skills and competence in the systems of care are mentioned in all nine articles. Providers were motivated to have more training to benefit persons with COP [47, 49, 51, 53], and the studies introduced ideas about training into reflective practices [48], local collaboration [52], community participation [54], a recovery orientation [50], meaningfulness and citizenship [55], and flexible approaches and skills for developing therapeutic alliances in secure physical milieus [47].

Generally, the articles argue that health outcomes of different types of housing need to be examined in research, including whether a client’s residency in a special category of housing can have long-term effects on substance use or the quality of life of tenants [48, 49]. A practical requirement was the sufficient resourcing of providers [48].

An important focus in Hansen’s study [48] was a form of supported housing that diverged from the original HF model described by Tsemberis (2010), which typically includes ACT teams with medical professionals such as doctors or psychologists. In contrast, the Norwegian teams followed the principles of Intensive Case Management, involving multidisciplinary teams composed of social workers, nurses, peer specialists, and other professionals. While the program did not fully implement the HFs principle of complete freedom of choice, it placed strong emphasis on respecting and acknowledging users’ perspectives and experiences. This approach encouraged joint reflection, enabling tenants to engage in deeper consideration before making significant life decisions. Service users were given a more central role in shaping their own support, while professionals acted as counsellors or facilitators rather than authoritative experts. Importantly, this user-centred approach helped professionals move beyond a purely diagnostic framework. By actively listening to participants and valuing their lived experiences, services became more personalized and responsive to individual needs. Although the model challenges the hegemony of professional knowledge, it does not fully realize the ideal of unrestricted choice. Nevertheless, Hansen concludes that Intensive Case Management, combined with joint reflection, contributes to more effective, user-oriented services and aligns with the broader shift in welfare systems from service delivery to co-production, where users and professionals collaborate in designing and delivering support [48].

The continuous evaluation of the success of the housing services in several areas listed below was conducted but not discussed in every article:

- Housing stability can be measured [49] and connected with feeling safe at home by keeping a stable housing irrespective of substance use [53];
- The measurement of substance use, which the service did not impact even though users wished for a reduction [49];
- Contact with service treatments impacted by a decrease of social services [49]; and
- The measurement of quality of life as an organisational tool, with official measures used [47–55] as reported in relation to the theme of mental health recovery.

### Societal responsibilities

The studies were contextualised differently. Five of the nine studies used national strategic guidelines, seven addressed the responsibilities of municipalities, three addressed the regional level or that of communities, and one primarily addressed the professional level (Table 5).

**Table 5:** Societal responsibilities

Authors	Year	Focus on responsibilities	Reasoning for housing policy	Need to solve	Housing principles and human rights	Impact of user-centredness on housing practices
Blid, et al.	2006	National level, Municipality, Municipal welfare boards	Community-based integrated living and housing interventions to promote inclusion in society.	Social integration	Evaluate degree of recovery and plan: continuum, integration of care, housing stability.	Everybodys’ right to access decent and affordable living in spite that tenants are likely to cause challenges, which need to be solved
Hansen, I	2018	National level, Municipality	Giving users a more prominent role in defining their services might lead to more effective service provision.	Respect and combatting stigma. Systemized user involvement.	Housing First as a fundamental right, a prerequisite for recovery. Harm reduction without sanctions.	Supporting the users’ choice and own recovery. Freedom of choice and self-determination require active engagement.
Lydahl & Lofstrand	2020	National level, Municipality, County council policies	The home turn represents a shift from paternalistic coercion to client-centredness and client choice.	Home as a human right.	Housing First principles: Housing and treatment should be separated.	Increasing the autonomy of tenants.
Matscheck, et al.	2019	National level, Regional level, Local level	The users perspective should be documented in the Coordinated Individual Plan.	Legislative questions.	Care coordination.	Users should know, who in the service is responsible for which needs.
Nesse, et al.	2020	National level, Municipality	Supported housing has a potential to promote recovery for persons with COP.	Financial challenges in arranging housing.	Developing a sense of home.	Tenant perceptions on being supported will enhance their strengths.
Nesse, et al.	2022	Municipality (local communities)	Humanistic values and attitudes in deinstitutionalization.	Developing community participation.	Developing meaningful activities.	Persons with COP may experience more difficulty accessing citizenship than others.
Nordaunet & Andvig	2018	Municipality	Own home is a human right and Housing First may stabilise everyday life.	Poverty and social exclusion.	Housing First is based on equality. Harm reduction, safety and keeping the home.	Tenants own preferences, own choices, the persons with COP are themselves responsible, they have resources and potential.
Ogundipe, et al.	2022	Municipality	Personal and social recovery are necessary for persons with COP	Promoting social community participation.	Recovery orientation.	Residents cannot participate in surrounding activities because of inadequate finances
Von Greiff, et al.	2020	Professionals	Recovery is influenced by internal, social and treatment-related factors.	Positive changes as a goal.	Changes and recovery require housing, employment and strengthening the social network.	Professionals need to break through client isolation and ‘learned passivity’ so that recovery may occur

Several articles refer to the deinstitutionalisation processes starting in the 1990s and the policies guaranteeing each citizen a home [49], which marked a major turning point in service provision that has shaped communities and even actual service encounters [51]. National guidelines regarding consumer choice and self-determination are challenging because power between professionals and vulnerable clients is not equally distributed, and municipalities may bear the responsibility of placing tenants in the housing facilities of their preference [48]. Meanwhile, self-governance may be expected of clients even amid challenging conditions such as COP [51]. According to Lydahl (2020) [51], any mental health service supported by ACT is fundamental for guaranteeing individuals with COP services on an equal basis [51]. By comparison, according to Nesse et al. (2020) [50], an integrated approach means that municipalities should assist individuals with COP in gaining access to adequate support [50]. A tool used in several Nordic countries is the coordinated individual plan, which is a requirement for providing sufficient support and coordinated care according to the needs and rights of persons with COP [52]. By contrast, Ogundipe et al. (2022) [54] investigated a service provided by an organisation that had not developed social inclusion and whose offerings were reduced due to a narrow biomedical model and dominant focus on economics [54].

In the Nordic countries, mainly city or municipality councils are responsible for implementing strategic housing guidelines, or else a specific municipal board manages the criteria for selecting housing services and/or policies for renting apartments [49–51]. A municipality or region, meanwhile, can choose the type of special-category housing. A major challenge is presented by policies for service organisation because social service authorities are responsible for housing, whereas treating mental health conditions within psychiatry is the responsibility of healthcare authorities [51]. Concerning organisational responsibility, tender processes may become more relevant than actual treatment or intervention, and economic constraints with reduced welfare budgets can impact users [49]. A good practice mentioned by Matscheck et al. (2019) [52] is a regional collaboration agreement covering both social services and healthcare; the agreement and the coordinated individual plan refine which authorities have responsibility and which interventions are planned for persons with COP [52]. At the clinical level, Matscheck et al. (2019) [52] also found that professional autonomy was restricted such that a long-term perspective could not be adopted, which resulted in coordinating and planning in the short term more than regional agreements would have allowed [52]. Another good practice mentioned by Nordaunet and Andvig (2018) was the use of a harm-reduction strategy [53]. If municipalities do not allocate resources for persons with COP, then tenants may be socially excluded, and their recovery may become restricted due to the absence of opportunities in their communities [54].

Last, questions that society needs to solve concern social integration [49, 54], combatting stigma [39], solving legislative dilemmas [44], and solving financial challenges in arranging housing [41].

### Ideals and values

The example HF prioritises creating housing stability, which appears to be seen as a fundamental right creating housing stability [48, 49, 53], as shown in Table 5. Community-based housing was augmented by including persons with COP into society [47–49, 54, 55] and within a shift from paternalism to emphasising client choice [51] via the process of deinstitutionalisation [55]. Some users were given a much stronger role that might have impeded more successful services [48, 53], and in that way, human rights and humanistic values had become intertwined with goals of personal or social recovery [47, 54].

Housing facilities can be regarded as a prerequisite for recovery [47, 48], perhaps because they can help to foster a sense of home [55] or become a basis for developing meaningful activities [55]. Within this context, individuality emerges as a dominant principle. Recovery is not treated as a one-size-fits-all process but is instead evaluated based on the individual’s needs and progress, which informs the planning of care pathways and strategies for social integration [49].

Harm reduction as a principle and ideal also impacted the types of interventions used [48], because even though everyone has a right to access adequate housing, persons with COP might present challenges for preventing harm [49]. Harm reduction as an approach was discussed in three studies, all aligned with the principles of HF [48, 51, 53]. Hansen et al. (2018) described a paradigm shift in Norwegian homelessness policy around the turn of the millennium, marking a transition from a “treatment first” model to a “housing first” strategy that prioritizes immediate access to housing, individualized follow-up services, and harm reduction [48]. A key feature of this approach is the absence of sanctions related to substance use, which was found to be instrumental in building trust, fostering cooperation, and enabling joint reflection between service users and providers [48].

Lydahl’s study highlighted that while harm reduction underpins the Housing First model, certain behavioural expectations remained in place—for example, clients were generally discouraged from smoking, drinking alcohol, or using drugs during home visits [51]. This reflects a negotiated balance between harm reduction principles and practical considerations in service delivery. Nordaunet and Andvig (2018) found that harm reduction in HF contributed to a more stable everyday life for participants, primarily through the security and autonomy associated with having one’s own home [53]. They added a holistic dimension to harm reduction, as housing safety was connected to both the autonomy of individuals, as to developing professional relationships, and the negotiation on the aims of the professional support.

Giving users a stronger voice can create new avenues for increasing policies of inclusion at the community level [48, 49]. Individual recovery is impacted by social and treatment-related factors [47], and humanistic values incorporating the right to an own home may also stabilise everyday life [53]. The general idea is that by increasing tenants’ autonomy [39, 42, 43] and increasing their responsibilities, they can use more of their own resources and potential [43].

### Active relationship building

A challenge presented by persons with COP is that though their individual choices and autonomy need to be developed, tapping into their resources and potential [42, 43] requires support from providers in developing meaningful activities [46].

Relationships between providers and service users are discussed in eight of the nine articles (Table 6), including in terms of the need to move away from paternalism in housing services for persons with COP [51]. At the same time, some studies on organising housing for such persons revealed the need to develop alliances and/or relationships between tenants and providers [37, 41] even despite financial constraints [45]. Some organisational guidelines are tailored towards persons with COP, including a prerequisite for starting in a housing programme of accepting follow-up services and home-based visits on a weekly basis [48]. Tenants may need providers’ support in keeping their housing and in utilising services within the larger organisation of services [53]. Nordaunet and Andvig (2018) have also suggested having tenants and providers create individualised plans for crises together [53]. Users themselves have the ultimate responsibility for their own lives, but providers are needed to create working alliances, especially in challenging situations in which they give hope to persons with COP [47].

**Table 6:** Practical work with persons with COP

Authors	Year	Intervention needs	Recovery orientation	Harm reduction	Health promotion, physical well-being	Floating & flexible service	Networking, collaboration	Individualised care	Family involved	Community participation	Meaningful activities	Peer support
Blid, et al.	2006	Solely housing is not sufficient. Interventions to deal with substance misuse and psychiatric problems.	No	No	No	No	No	Yes	No	No	No	No
Hansen, I	2018	Staff is needed to develop users’ choice by contributing with reflective skills such as advice, guidance, questions and support.	Yes	Yes	No	Yes	Yes	Yes	No	Yes	No	No
Lydahl & Lofstrand	2020	Doing good care: Using relationships to negotiate and build what is good for the client.	Yes	Yes	No	Yes	Yes	Yes	Yes	Yes	Yes	No
Matscheck, et al.	2019	Multidisciplinary teams providing integration to help by a Coordinated Individual Plan.	No	No	No	No	Yes	Yes	No	No	No	No
Nesse, et al.	2020	Staff are needed to support recovery in supported housing,	Yes	No	No	No	No	Yes	Yes	Yes	Yes	No
Nesse, et al.	2022	Activities and occupations that give structure to everyday life.	Yes	No	No	No	No	Yes	Yes	Yes	Yes	No
Nordaunet & Andvig	2018	Workers show respect and warmth, and work together with the person with COP as long as necessary.	Yes	Yes	Yes	Yes	No	Yes	Yes	Yes	Yes	No
Ogundipe, et al.	2022	Funding for leisure would support participation in society	Yes	No	No	No	No	No	No	Yes	Yes	Yes
Von Greiff, et al.	2020	More extensive system of support with interventions focusing on specific problems, regular treatment contacts.	Yes	No	No	Yes	Yes	Yes	No	Yes	Yes	Yes

Hansen et al. (2018) have suggested joint reflective practices to help users to express their opinions and needs. For professionals engaging the users in co-productive work, such practices mean setting aside their professional knowledge and hegemony and focusing on listening and identifying freedom of choice for users [48]. Even though there is an imbalance in power distribution, the professionals had expertise in areas that helped users to make choices about managing their personal finances [48]. At the same time, persons without the skills in such everyday practices might not be allowed to make individual decisions towards gaining mastery over choices in their lives [51].

### Recovery, positive mental health

Practices supporting recovery were found to be organised according to various treatment principles (Table 6). For persons with COP, housing or supported housing were not sufficient, because they needed supportive interventions for both substance use problems and mental health conditions [49]. Given their vulnerabilities, they thus needed specialised professional support [47, 48, 50–53]. There were only a few examples of services with untrained providers, but there appeared to be challenges in delivering such services [54].

An individualised treatment approach was used in eight of nine studies in the articles reviewed, which reflects the ideals and values of the Nordic countries [46]. Being oriented towards recovery and adopting a positive mental health approach were adopted in seven studies, as reflected in the general background on articles about persons with COP [4, 6, 27, 30]. A community approach with some aim at inclusion in society was adopted in another seven articles, and developing meaningful activities in everyday life was adopted in six studies. Meanwhile, the matter of inclusion in the sense that abstinence is not a requirement, nor is harm reduction, was found in three studies. Beyond that, suggestions for floating and flexible services were addressed in four studies, networking and collaboration in another four studies, and family involvement in yet another four articles, whereas health promotion, physical well-being, and peer support were rarely addressed. Notably, peer support—widely endorsed in mental health and substance use treatment—was only briefly mentioned in two studies [47, 54](Table 6).

Despite being a measurable outcome, quality of life can be defined in various ways: as staying in a programme [49, 53], whether the users could impact their choices within the housing programmes [48, 51], the amount of individual variation available [52], a recovery orientation [47, 50, 54, 55], life satisfaction [50], the meaningfulness of daily activities [55], participation and developing social relationships [47, 54], and regularly evaluated individualised care plans [52]. We interpreted all of those definitions as indicating a goal towards contentment in life.

## Discussion

Focusing on the Nordic context, we reviewed literature to identify research gaps in the areas of organisation, coordination, and networking of housing and service provision for people with COP, and we explored the research-based recommendations for organising and coordinating housing services for persons with COP. As a result, we identified the importance of organising housing together with other services using a universal multidisciplinary lens and the fundamental right of having a home.

Our results show that the housing and service options for the most vulnerable individuals—that is, with long-term, complex, severe mental ill health and substance issues—have limited research evidence for informing practice in the field. The Nordic perspective was limited to research from Norway and Sweden, with studies on housing from Denmark and Finland focusing on either severe mental illness or substances, and no studies originating from Iceland. We started the review with a Nordic perspective, but ended up with studies from solely two Nordic countries. There is a lack of research on the topic because it is very difficult to study, and because of the complexities involved, or there may exist stigma deterring research within this complex area, which may explain the continued marginalisation of persons with COP. Our approach included the community level in our original search, and we found that persons with COP have to endure societal stigma and that their integration in society is not a straightforward step. Stigma within society on mental health and especially persons with severe COP may distract persons with COP from receiving the support that they need and drive social exclusion, as Tweed et al. (2021) have noted [56]. On the contrary, there is a need to develop practices for promoting the inclusion of persons with COP within societies and communities. National policy recommendations and actual regional and municipal decisions on implementation need to support approaches that promote inclusion. Integrated, effective care requires a cultural shift, as Harris et al. (2023) have shown [3]. Regions also need mechanisms of governance to monitor progress in implementing policy, as Wiktorowitcz et al. (2019) have observed [13].

In the Nordic context, the organisation of the public sector is responsible for managing public health, and mental healthcare within healthcare, substance services mainly within social services, and the entire network of specialised services. Because persons with COP are among the most vulnerable groups in the housing market [47, 50] and are likely to experience adverse living conditions [54], the Nordic countries seem to aim to include each citizen, in contrast to the possible exclusion of persons with COP as Tweed et al. (2021) have observed [56]. Hansen (2018) has suggested incorporating more mental health support (ACT) within the organisation of housing services and facilities, which aligns with previous findings concerning persons with co-occurring disorders [7, 13, 23]. A key feature in three of the housing approaches within harm reduction was the absence of sanctions related to substance use, and added with the need of building relationships, trust, fostering cooperation, and enabling joint reflection between service users and providers [8, 48, 51, 53]. This reflects a negotiated balance between harm reduction principles and practical considerations in service delivery, and on the other hand the complexity within the clinical level, addressing the core necessity for care integration also within housing for persons with COP. We can identify a need for more research in the area of implementation harm reduction strategies within housing and HF for persons with COP.

The recovery orientation was mentioned in seven of nine Nordic studies in our review (Table 6). In our study, eight of the studies focused on individualised support (Table 6) and the fundamental understanding of change as being person-driven [6, 57], similarly to what Nordic guidelines mention [46]. As suggested by Weiss et al. (2016), the recovery orientation and health-promoting approach on a community level should be systematically inclusive across sectors and support people in taking control of their own health [27]. Topor et al. (2022) have emphasised that “*recovery is a deeply social, unique, and shared process in which our living conditions, material surroundings, social relations and sense of self evolve*” (p. 11) [6]. The move within psychiatry and health systems from institutions and at times following deinstitutionalisation has also meant a shift towards humane values of care and policies. Topor et al. (2022) sees the recovery movement as a social change geared towards ensuring each citizen the possibility “*to live satisfying, hopeful, and reciprocal lives, even though we may still experience threats, stressful social situations, and distress*” (p. 11) [6]. We think that engagement in any encounter is important for developing relationships, new ways of meaning making, and that understanding and handling one’s life situation can be jointly co-created.

In our study, we identified the need of organizing services as a whole (Table 4), the different levels of societal responsibilities (Table 5), and many practical aspects of housing organisation and networking affecting persons with COP (Table 6). Based on international research on Dual Disorder Treatment, Dual Disorder housing and integrated care guidelines [3, 17, 38, 58], we could identify that the included research studies incorporate a micro, meso and macro dimension of service organisation, coordination, and networking of housing and service provision as suggested by van Hoorn et al. (2024) [33]. The clinical (meso) level is in line with the person-centred, recovery-oriented clinical level of care coordination that we identified. The multidisciplinary character of praxis requires professional integration (meso), as well as developing inter-organizational relationships (meso) to reach organizational integration. Our study has also pointed out that persons with COP need a multitude of services alongside housing facilities, which is a challenge for service organisations and interagency collaboration among service providers.

Although providing housing can improve housing stability, improving social integration requires housing services to take into account COP and the individuals as whole human beings and to address their needs [13]. That need aligns with previous studies from other Western countries that have stressed ideals of support for persons with COP [3–6], who may have a history of social isolation and do not necessarily possess the skills needed to participate in daily social activities. Housing stability can improve life satisfaction, but without support in areas such as participation in such activities, persons with COP might experience discontentment, a lack of belonging, and a sense of meaninglessness [41], all of which may entrench their social exclusion. Some of the studies expressed that regional economics and budgeting were considered to be a restriction on building a practical level of recovery and activities in everyday life [23, 54]. However, persons with COP are entitled to care, interventions, activities and treatment as a human right, as recent research has underscored [59]. We want to repeat the importance of basic values guiding the practices: the right to adequate housing, is internationally recognised in the UN Universal Declaration of Human Rights (1948) [19]. We agree with van Hoorn et al. (2024) of the need of unifying the service system dimensions via normative and functional integration to benefit the persons with COP by seeing homes and housing as a human right [33]. High quality community-based mental health care protects human rights, and supports users in their recovery within a network in the community [28]. In our review themes the positive mental health and recovery orientation together with building relationships are indicators of the inclusive ideals and values in the two Nordic countries.

One challenge in particular arises from the fact that persons with COP receive support and help from different disciplinary systems and organisations of treatment [60]. If local authorities and organisations do not coordinate the system—for example, via multidisciplinary teamwork, case management, or individual care plans, as a Swedish example [52] has shown and strategic guidelines in Norway support [61], the worst-case scenario is that persons with COP are excluded from receiving help. System integration (macro) would require horizontal and vertical system integration [33]. An organisational systems perspective requires consideration of the ways in which processes and outcomes within a system drive change [45]. Emphasising primary care and public health was not as evident in our review of articles from the Nordic countries, in contrast to Nordic strategic guidelines, which have a strong focus on public health approaches for helping persons with COP [46], with an emphasis on specialised skills, knowledge, and competence within primary care. Only one of the articles included the physical well-being of persons with COP, which is quite worrying considering that persons with COP have a far lower life expectancy in the Western world [9, 10] and are at risk of diagnostic overshadowing, meaning that their somatic and medical symptoms are misinterpreted as symptoms of mental illness and distress [12]. There is a lack of research in the area of housing, persons with COP and using primary care facilities.

For the different levels to optimally support the user, those need to be structured around the primary process of service delivery [33], and make use of interventions based on user goals [28]. It may be impossible for single municipalities to undertake the demanding task of producing holistic housing and interventions for persons with COP. The Swedish strategic guidelines [62] suggest considering collaboration between several municipalities [46]. Another solution would be to arrange services at the regional level. On the other hand, municipal-level housing is most relevant for persons with COP, who thus do not need to move to another municipality because the housing is jointly arranged.

Persons with COP should receive sufficient integrated support [1, 3, 4, 13, 17, 56, 60], as made evident in the articles that we reviewed. Integrated care is an approach to overcome care fragmentation [32–34]. From the perspective of promoting mental health, integrating a care model means that there should be no wrong paths forward for clients and should involve a universal, person-centred, equity-based approach [3, 14, 17]. It may be that supporting the most challenging persons with COP in a trauma-informed way, has not yet been implemented in primary or mental healthcare even in the Nordic countries, which is in line with Chaudhri et al. (2019) notions from the criminal justice system [16].

A challenge in evaluating services is that national policy authorities may recommend or require the constant improvement of individuals in the housing facilities based on evaluations requiring a multitude of longitudinal data, even though persons with COP may not recover as the general population does or demonstrate required elements of recovery [6, 30]. Persons with COP need unique evaluation tools because their mental health, substance problems and/or social integration as tenants may not improve, as Hansen (2018) has noted [48]. If the service organisation and local municipality or regional authorities cannot demonstrate improvement, then the question becomes whether the policies are sustainable enough for the scope of COP so that tenants can keep their homes even when no clear improvement has occurred? Within services for COP, harm-reduction strategies, the recovery movement, and HF emphasise increasing individual choice and do not require full abstinence to maintain housing. The approach ensures that housing and treatment are provided regardless of sobriety. From that perspective, more research is needed on how those principles function together, and regardless of improvement, human rights entitle a home for everyone. The matter of social justice needs to be taken seriously within policies and those authorities, who allocate resources. Our questions within the complexities are, who is evaluating the needs and levels of support within housing for persons with COP? There are vast skill requirements for such tasks, also in order to support the self-determination of persons with COP.

In the context of COP, it is essential to recognise that evidence extends beyond conventional research data to include insights derived from clinical practice and the lived experiences of patients. Implementing national health policy guidelines to build capacities in order to achieve the desired outcomes is primarily up to the regions and municipalities in the Nordic countries even though they may lack resources and time [27]. Traditionally, EBP has prioritised quantitative methodologies—most notably, randomised controlled trials (RCTs)—as the ‘gold standard’ for generating valid evidence [63]. However, this methodological emphasis often marginalises qualitative research and other forms of systematic experiential knowledge, potentially resulting in a fragmented or biased comprehension of the complex health issues for persons with COP. In alignment with scholars such as Greenhalgh (2014) [53] and Weiss et al. (2016) [12], we advocate for a broader conceptualisation of evidence that embraces methodological pluralism. A limited capacity to critically assess and apply diverse forms of evidence may hinder knowledge-informed decision-making [12]. While neither randomized controlled trials (RCTs) nor qualitative research alone can fully capture the complexity of challenges in health and social care, we argue that recognizing and legitimizing the value of qualitative research is essential for gaining a more comprehensive understanding of how integrated housing services are organized and coordinated for persons with COP. In line with Frost et al., (2017) we underscore the importance of methodological diversity and the need of multidimensional evaluations in capturing the complexity of integrated care services [29].

However, complexity in problems and fragmentation of services can often hinder consistent application of best practices and implementation. Implementation science becomes a critical tool for translating recovery-oriented models into everyday practice, as proposed by Frost et al. (2017) [29] and THCS (2023) [64]. Additionally, improvement science primarily examining the systematic integration of evidence or knowledge-based interventions into real-world settings could enhance the quality and performance within healthcare systems [64]. The importance of adaptive, context-sensitive implementation strategies that align with local needs is highlighted by Looman et al. 2021 as essential for ensuring sustainable and effective implementation of integrated care [32]. The healthcare systems aim at avoiding fragmentation and increasing the sustainability by means of integration [34].

There are at least four models for developing complex systems. The mental health ecosystems approach may yield value in developing public services, as it takes a whole-systems approach to mental healthcare and analyses local contextual and environmental data, for implementation and translation into policy and practice [65]. The organisation and management level of services, the service praxis level, and the institutional organisations and networks, need to take into consideration individual and belief levels [66]. Secondly, from an integration perspective, the clinical (micro), professional, and organizational (meso), and system (macro) levels form needs and complexities, but include tools to overcome fragmentation [33]. Thirdly, the SELFIE Framework for Integrated Care for multi-morbidity frames the individual with multi-morbidities at the core, and calls for flexibility within the current structures [32]. The COSMHAD framework aims at enhancing the outcomes, and include a whole-person approach, staff competencies, committed leadership and policies to support co-occurring disorders [3]. The four approaches differ in the conceptualisation of the complexities and the service system dimensions. Looman et al. (2021) discuss the environment, service delivery, leadership and governance, workforce, financing, technologies and medical products, and information and research [32].

Within mental health ecosystems the macro-level includes the institutional level, societal norms and rules, and the service level is the meso-level, whereas individuals such as staff and users are perceived as the micro-level, and beliefs are a sub-micro-level [66]. There is a need for more research within the frameworks of integration, utilising a bottom-up approach enhancing the core value of achieving the best possible outcomes for the service users, and to enable organizations to achieve sustainable healthcare services [33]. It seems that the success of these processes on different levels would need to rely on dynamic interactions across organisations, societies, networks, and individual subsystems [66], and the diverse levels require distinct implementation strategies [32]. Our review sheds light on the interactions in between the diverse levels for housing for persons with COP. In the future, it is necessary to define the distinct service system levels or dimensions more clearly. When planning the organisation of services for persons with COP, their individuality and the micro-level perspective need to be considered as the basis for searching for solutions at the macro level, including the adequacy of housing [49]. Adjusting to the needs of persons with COP will probably benefit them and society, which can enhance their options for integrating into society. Our study showed that recovery-orientation and reinforcing hope are essential in the housing services for persons with COP, and we agree that the development of regional mental health models are recommended, as suggested by Frost et al. (2017) [29]. There is a need to develop specified guidelines on integration within housing services and the coordination and networking for persons with COP.

We advise municipalities and regions to offer individualised support options for persons with COP along with housing within a harm-reduction frame, even if it creates challenges regarding the provider’s professional skills and competencies. Regions could develop their areas in regard to normative integration meaning development and maintaining a common frame between individuals, professional groups and individuals on the micro, meso and macro levels [33]. Providers working with persons with COP inevitably need special skills such as incorporating flexibility, ACT, or case management and transdisciplinary work. From the providers’ perspective, research shows that working in an intensive way with the persons with COP may also include challenges and create crisis situations and even predispose providers to dangers in their work environment, which is why safety in work environments needs to be addressed [59]. Because many nongovernmental housing services are delivered without providers [54] and via peer support, an emerging question concerns the role of the development of the workforce competencies, for it also ensures that persons with COP receive adequate support. The needs for health promotion and the physical well-being of tenants were mentioned in one study that we reviewed, and previous research has also emphasised that point. Additionally, service users and their peers should be actively involved in service design [28]. It is essential to integrate and enhance the role of service users and peers across the micro, meso and macro levels.

Complex practices that involve several interconnected actors, levels, activities, and elements are suggested to be developed in interdisciplinary collaboration and in client-centred, culturally sensitive ways [28, 29, 64]. By employing a holistic and human rights-based approach that integrates diverse forms of evidence, decision-making processes can be grounded in comprehensive, multifaceted information, thereby ensuring that interventions and strategies are both evidence-based and precisely tailored to address the needs of service users and other key stakeholders. Developing such complex practices can occur via implementation using established solutions developed within practice over time and indicate an opportunity for such practices to develop solutions within healthcare and social care [64].

### Limitations and strengths

We opted to conduct a scoping review because of the complexity and breadth of our research interest [32] and the need to thoroughly discuss the concepts being investigated. Methodologically speaking, the search was completed within internationally well recognised databases in collaboration with and under the guidance of a search informatics specialist. We screened many databases and included a wide set of search terms, which are openly available in Supplementary File 1. Two blinded authors screened the references in Rayyan, and we also completed a quality appraisal of the studies blinded by another two authors. We additionally followed the PRISMA-ScR Checklist [41] at all stages of the study, and the process is openly available to readers. It was a surprise that we could only identify 9 articles from the Nordic countries within the context of organisation of housing services for persons with COP. The reason was that so many housing articles focused on SMI, and did not include persons with COP. This means that there is a need for Nordic studies incorporating the housing and COP perspective.

A solution might have been to first analyse the studies with solely a mental ill health and housing approach, and secondly the set of studies we focused on with persons with COP. This way we would have been able to identify a broader material for analysis.

We have observed extreme complexity within the organisation of services, even though reporting will always seem somewhat simplified. The holistic approach that we utilised raises new questions about the well-being of persons with COP.

A strength is that the Nordic countries definition incorporated all Nordic countries (Denmark, Finland, Iceland, Norway and Sweden), and not solely Scandinavia (Denmark, Norway and Sweden). A reason why we might not have found more research on housing organisation and networking for persons with COP is that the guidelines of publishers usually refine the length and focus of articles. Additionally, we observed in our research that ethical committees may be very strict regarding vulnerable populations. A risk is that sensitive questions concerning SMI and problematic substance use are not allowed, and research among the most complex and vulnerable citizens might have been restricted.

Because our research interest was so complex, the search terms may have limited the results, and we searched for both “severe mental illness”, “severe substance use”, and “housing” (see Supplementary File 1). Other reviewers in the area have also expressed similar challenges, and we had to make selections based on our research interests. Grey literature could have included interesting data, but it was very challenging to use the current grey literature databases to search for answers to such complex questions.

Our analysis focused on research articles, but another review of policy recommendations and guidelines is in progress [46]. Because both works take a practical viewpoint on organising services at a local or regional level, the two articles will complement each other. Because of the scarcity of studies, this article does not give a thorough analysis of the service systems within the Nordic countries. Because a Nordic review has never before been conducted in the field of housing and organisation and coordination of services for persons with COP, our study gives new information on the practice in the field in the Nordic countries and informs other regions as well of challenges in high-income settings. Because of lack of research, we could not identify clear cultural differences between the countries.

## Conclusions and implications for research and practice

Our results, shown in Fig. 2, describe a model in which all aspects bring significant elements to the housing and organisation of services for persons with COP. We adopted a Nordic perspective with unique cultural values of inclusion, specific welfare systems, policy frameworks, and geographical considerations. Ideals and values include humanistic values with a user orientation and a strong rights-based approach. The recommendations for the organisation and coordination of services for persons with COP include ensuring the right to a home, ensuring social integration and community involvement, combatting stigma, and systemising user involvement. The approach of emphasizing primary care and multidisciplinary mental health is yet today a challenge even in high-income areas. The integration of services was not on an ideal level, even though the housing policies were human rights-based. We identified societal responsibilities stemming from national strategies ensuring each citizen adequate support and inclusion in society, and the necessity of harm reduction principles within housing. It is possible that functional integration does not take place in all regions. As the working methods were based on active relationship building to be able to help persons with COP, we interpreted this as a sign of clinical integration. Our findings can contribute to the discussion on organising housing services in light of complexity to persons with COP. There are research gaps in all Nordic countries and the research within housing for persons with COP is scarce. We call for using multi-dimensional research approaches.

The implications for policymakers and practitioners based on our scoping review are as follows:

- Persons with COP need their own homes, which can be regarded as a basic human right and means of allowing a dignified life for everyone. Ensuring the active engagement of tenants is a way to ensure both personal and social recovery.
- Persons with co-occurring conditions often face barriers to healthcare and housing, which requires comprehensive and accessible health services that lower barriers to support.
- To ensure competencies, service providers well-trained in addressing co-occurring disorders are critical for delivering effective care.
- Services for COP need to involve comprehensive EBP that address both immediate needs and long-term recovery.
- Research in marginalised and vulnerable populations is an ethical commitment, why more research investments are required.

## Electronic supplementary material

Below is the link to the electronic supplementary material.

Supplementary Material 1

Supplementary Material 2

## Data Availability

Data sharing is not applicable to this article as no datasets were generated or analysed during the current study. The data that support the findings of this study are available from the first authors upon reasonable request.

## Abbreviations

ACT: Assertive Community Treatment
EBP: Evidence Based Practice
COP: Persons with co-occurring substance use and mental health problems
HF: Housing First
JBI: Joanna Briggs Institute
TF: Treatment First
SMI: Severe Mental Illness

## References

[1] Hakobyan S, Vazirian S, Lee-Cheong S, Krausz M, Honer WG, Schutz CG. Concurrent disorder management guidelines. Systematic review. J Clin Med. 2020;9(8). 10.3390/jcm9082406 PMC7463987 32731398

[2] Keyser DJ, Watkins KE, Vilamovska AM, Pincus HA. Improving service delivery for individuals with co-occurring disorders: new perspectives on the quadrant model. Psychiatr Serv. 2008;59(11):1251–53. 18971399 10.1176/ps.2008.59.11.1251 PMC6545908

[3] Harris J, Dalkin S, Jones L, Ainscough T, Maden M, Bate A, et al. Achieving integrated treatment: a realist synthesis of service models and systems for co-existing serious mental health and substance use conditions. Lancet Psychiatry. 2023;10(8):632–43. 37327804 10.1016/S2215-0366(23)00104-9

[4] Thornicroft G, Deb T, Henderson C. Community mental health care worldwide: current status and further developments. World Psychiatry. 2016;15(3):276–86. 27717265 10.1002/wps.20349 PMC5032514

[5] Patel V, Saxena S, Lund C, Thornicroft G, Baingana F, Bolton P, et al. The Lancet Commission on global mental health and sustainable development. Lancet. 2018;392(10157):1553–98. 30314863 10.1016/S0140-6736(18)31612-X

[6] Topor AP, Bøe TD, Larsen IB. The lost social context of recovery psychiatrization of a social process. 2022. 10.3389/fsoc.2022.832201 PMC9022098 35463189

[7] Miller JA, Carver H, Masterton W, Parkes T, Maden M, Jones L, et al. What treatment and services are effective for people who are homeless and use drugs? A systematic ‘review of reviews’. PLoS ONE. 2021;16(7). 10.1371/journal.pone.0254729 PMC8279330 34260656

[8] Watson DP, Orwat J, Wagner DE, Shuman V, Tolliver R. The housing first model (HFM) fidelity index: designing and testing a tool for measuring integrity of housing programs that serve active substance users. Subst Abuse Treat Prev Policy. 2013;8(1):16–. 23641860 10.1186/1747-597X-8-16 PMC3655861

[9] Nordentoft M, Wahlbeck K, Hällgren J, Westman J, Osby U, Alinaghizadeh H, et al. Excess mortality, causes of death and life expectancy in 270,770 patients with recent onset of mental disorders in Denmark, Finland and Sweden. PLoS ONE. 2013;8(1):e55176. 23372832 10.1371/journal.pone.0055176 PMC3555866

[10] Heiberg IH, Jacobsen BK, Nesvag R, Bramness JG, Reichborn-Kjennerud T, Naess O, et al. Total and cause-specific standardized mortality ratios in patients with schizophrenia and/or substance use disorder. PLoS ONE. 2018;13(8):e0202028. 30138449 10.1371/journal.pone.0202028 PMC6107156

[11] Martens N, De Haeck E, Van De Vondel E, Destoop M, Catthoor K, Dom G, et al. Physical healthcare for people with a severe mental illness in Belgium by long-term community mental health outreach teams: a qualitative descriptive study on physicians’, community mental health workers’ and patients’ perspectives. Int J Environ Res Public Health. 2023;20(1):811. 36613132 10.3390/ijerph20010811 PMC9819842

[12] Molloy R, Brand G, Munro I, Pope N. Seeing the complete picture: a systematic review of mental health consumer and health professional experiences of diagnostic overshadowing. J Educ Chang Clin Nurs. 2023;32(9–10):1662–73. 10.1111/jocn.16151 34873769

[13] Wiktorowicz M, Abdulle A, Di Pierdomenico K, Boamah SA. Models of concurrent disorder service: policy, coordination, and access to care. Front Psychiatry. 2019;10:61. 30837903 10.3389/fpsyt.2019.00061 PMC6389671

[14] Sorsa M, Greacen T, Lehto J, Åstedt-Kurki P. A qualitative study of barriers to care for people with co-occurring disorders. Arch Psychiatr Nurs. 2017;31(4):399–406. 28693877 10.1016/j.apnu.2017.04.013

[15] McDonell MG, Kerbrat AH, Comtois KA, Russo J, Lowe JM, Ries RK. Validation of the co-occurring disorder quadrant model. J Psychoact Drugs. 2012;44(3):266–73. 10.1080/02791072.2012.705065 23061327

[16] Chaudhri S, Zweig KC, Hebbar P, Angell S, Vasan A. Trauma-informed care: a strategy to improve primary healthcare engagement for persons with criminal justice system involvement. J Gen Intern Med. 2019;34(6):1048–52. 30912031 10.1007/s11606-018-4783-1 PMC6544694

[17] Minkoff K, Covell NH. Recommendations for integrated systems and services for people with co-occurring mental health and substance use conditions. Psychiatr Serv. 2022;73(6):686–89. 34644127 10.1176/appi.ps.202000839

[18] McGovern MP, Clark RE, Samnaliev M. Co-occurring psychiatric and substance use disorders: a multistate feasibility study of the quadrant model. Psychiatr Serv. 2007;58(7):949–54. 17602011 10.1176/ps.2007.58.7.949

[19] The right to adequate housing. In Habitat U, editor. 21 edn. Geneva: office of the United Nations High Commissioner for Human Rights; 2010.

[20] Tsemberis S, Gulcur L, Nakae M. Housing first, consumer choice, and harm reduction for homeless individuals with a dual diagnosis. Am J Public Health. 2004;94(4):651–56. 15054020 10.2105/ajph.94.4.651 PMC1448313

[21] Padgett KD, Henwood FB. Qualitative research for and in practice: findings from studies with homeless adults who have serious mental illness and co-occurring substance abuse. Clin Soc Work J. 2012;40(2):187–93.

[22] Nelson G, Laurier W. Housing for people with serious mental illness: approaches, evidence, and transformative change. J Sociol Soc Welf. 2010;XXXVII(4):123–46.

[23] Hammervold U, Gytri S, Storm M, Gilje Lid T, Sagvaag H. What is known about persons with co-occurring problems’ experiences with supported housing, recovery, and health promotion? A scoping review. Draft. 2024. 10.1186/s12913-024-11736-z PMC11549848 39516887

[24] Salyers MP, Tsemberis S. ACT and recovery: integrating evidence-based practice and recovery orientation on assertive community treatment teams. Community Ment Health J. 2007;43(6):619–41. 17514503 10.1007/s10597-007-9088-5

[25] Allred CA, Burns BJ, Phillips SD. The assertive community treatment team as a complex dynamic system of care. Adm Policy Ment Health. 2005;32(3):211–20. 15844845 10.1007/s10488-004-0841-6

[26] Davidson L, Andres-Hyman R, Bedregal L, Tondora J, Frey J, Kirk TA. From “double trouble” to “dual recovery”: integrating models of recovery in addiction and mental health. J Dual Diagn. 2008;4(3):273–90.

[27] Weiss D, Lillefjell M, Magnus E. Facilitators for the development and implementation of health promoting policy and programs - a scoping review at the local community level. BMC Public Health. 2016;16:140. 26869177 10.1186/s12889-016-2811-9 PMC4751684

[28] Keet R, de Vetten-Mc Mahon M, Shields-Zeeman L, Ruud T, van Weeghel J, Bahler M, et al. Recovery for all in the community; position paper on principles and key elements of community-based mental health care. BMC Psychiatry. 2019;19(1):174–11. 31182058 10.1186/s12888-019-2162-z PMC6558752

[29] Frost BG, Tirupati S, Johnston S, Turrell M, Lewin TJ, Sly KA, et al. An Integrated Recovery-oriented Model (IRM) for mental health services: evolution and challenges. BMC Psychiatry. 2017;17(1):22–. 28095811 10.1186/s12888-016-1164-3 PMC5240195

[30] Leamy M, Bird V, Boutillier CL, Williams J, Slade M. Conceptual framework for personal recovery in mental health: systematic review and narrative synthesis. Br J Psychiatry. 2011;199(6):445–52. 22130746 10.1192/bjp.bp.110.083733

[31] Storm M, Fortuna KL, Gill EA, Pincus HA, Bruce ML, Bartels SJ. Coordination of services for people with serious mental illness and general medical conditions: perspectives from rural Northeastern United States. Psychiatr Rehabil J. 2020;43(3):234–43. 31985242 10.1037/prj0000404 PMC7382986

[32] Looman W, Struckmann V, Köppen J, Baltaxe E, Czypionka T, Huic M, et al. Drivers of successful implementation of integrated care for multi-morbidity: mechanisms identified in 17 case studies from 8 European countries. Soc Sci Med. 2021;277:113728. 33878666 10.1016/j.socscimed.2021.113728

[33] van Hoorn ES, Ye L, van Leeuwen N, Raat H, Lingsma HF. Value-based integrated care: a systematic literature review. Int J Health Policy Manag. 2024;13(1):8038. 38618830 10.34172/ijhpm.2024.8038 PMC11016279

[34] Piquer-Martinez C, Urionagu A, Benrimoj SI, Calvo B, Dineen-Griffin S, Garcia-Cardenas V, et al. Theories, models and frameworks for health systems integration. A scoping review. Health Policy. 2024;141:104997. 10.1016/j.healthpol.2024.104997 38246048

[35] Alsuhaibani R, Smith CD, Lowrie R, Aljhani S, Paudyal V. Scope, quality and inclusivity of international clinical guidelines on mental health and substance abuse in relation to dual diagnosis, social and community outcomes: a systematic review. BMC Psychiatry. 2021;21(1). 10.1186/s12888-021-03188-0 PMC8066498 33892659

[36] Bjerge B, Houborg E, Edman J, Perälä R. Concepts and policies directed at drug use in Denmark, Finland, and Sweden. In: Hellman M, Berridge V, Duke K, Mold A, editors. Concepts of addictive substances and behaviours across time and place. Oxford Academic; 2016. p. 33–56.

[37] Nesvaag S, Lie T. The Norwegian substance treatment reform: between new public management and conditions for good practice. Nordisk Alkohol Nark. 2010;27(6):655–66.

[38] Miler JA, Foster R, Hnizdilova K, Murdoch H, Parkes T. ‘It maybe doesn’t seem much, but to me it’s my kingdom’: staff and client experiences of Housing First in Scotland. Drugs: Educ Prev Policy. 2022;29(3):231–44.

[39] Peters MD, Godfrey CM, Khalil H, McInerney P, Parker D, Soares CB. Guidance for conducting systematic scoping reviews. Int J Evid Based Healthc. 2015;13(3):141–46. 26134548 10.1097/XEB.0000000000000050

[40] Munn Z, Peters MDJ, Stern C, Tufanaru C, McArthur A, Aromataris E. Systematic review or scoping review? Guidance for authors when choosing between a systematic or scoping review approach. BMC Med Res Methodol. 2018;18(1):143–. 30453902 10.1186/s12874-018-0611-x PMC6245623

[41] Tricco AC, Lillie E, Zarin W, O’Brien KK, Colquhoun H, Levac D, et al. PRISMA extension for scoping reviews (PRISMA-ScR): checklist and explanation. Ann Intern Med. 2018;169(7):467–73. 30178033 10.7326/M18-0850

[42] Moher D, Shamseer L, Clarke M, Ghersi D, Liberati A, Petticrew M, et al. Preferred reporting items for systematic review and meta-analysis protocols (PRISMA-P) 2015 statement. Systematic Rev. 2015;4(1):1–9. 10.1186/2046-4053-4-1 PMC4320440 25554246

[43] Institute JB. Critical appraisal tools, Joanna Briggs Institute. 2024. https://jbi.global/critical-appraisal-tools.

[44] Page MJ, McKenzie JE, Bossuyt PM, Boutron I, Hoffmann TC, Mulrow CD, et al. The PRISMA 2020 statement: an updated guideline for reporting systematic reviews. BMJ. 2021;372:n71. 33782057 10.1136/bmj.n71 PMC8005924

[45] Petticrew M, Knai C, Thomas J, Rehfuess E, Noyes J, Gerhardus A, et al. Implications of a complexity perspective for systematic reviews and guideline development in health decision making. BMJ Global Health. 2019;4. 10.1136/bmjgh-2018-000899 PMC6350708 30775017

[46] Sorsa M, Hummervold U, Benedixen A, Sagvaag H. The organization of housing and service provision for persons with co-occurring substance use and mental health problems. A documentary analysis of nordic strategic guidelines in ROP Municipal. Draft. 2024.

[47] Von Greiff N, Skogens L, Topor A. Supporting recovery in social work with persons having co-occurring problems - clients’ and professionals’ perceptions. Nord Soc Work Res. 2020;10(2):173–85.

[48] Hansen ILS. Users’ choice in providing services to the most vulnerable homeless people. Soc Incl. 2018;6(3):319–26.

[49] Mats B, Arne G. Socially excluding housing support to homeless substance misusers: two Swedish case studies of special category housing. Int J Soc Welf. 2006;15(2):162–71.

[50] Nesse L, Gonzalez MT, Aamodt G, Raanaas RK. Recovery, quality of life and issues in supported housing among residents with co-occurring problems: a cross-sectional study. Adv Dual Diagn. 2020;13(2):73–87.

[51] Lydahl D, Lofstrand CH. Doing good: autonomy in the margins of welfare. Sociol Health Ill. 2020;42(4):892–906. 10.1111/1467-9566.13069 32115744

[52] Matscheck D, Piuva K, Eriksson L, Åberg M. The coordinated individual plan - is this a solution for complex organizations to handle complex needs? Nord Soc Work Res. 2019;9(1):55–71.

[53] Nordaunet OM, Andvig E. Møte mellom skadereduksjon og Housing First i Norge – ansattes beskrivelser. Tidsskr for psykisk helsearbeid. 2018;15(1):52–62.

[54] Ogundipe E, Sælør KT, Biong SU. Social recovery and economy: a thematic analysis of staffs’ experiences with promoting social community participation for residents with co-occurring problems. Adv Dual Diagn. 2022;15(1):37–50.

[55] Nesse L, Aamodt G, Gonzalez MT, Rowe M, Raanaas RK, et al. The role of occupational meaningfulness and citizenship as mediators between occupational status and recovery: a cross-sectional study among residents with co-occurring problems. Adv Dual Diagn. 2022;15(3):99–118.

[56] Tweed EJ, Thomson RM, Lewer D, Sumpter C, Kirolos A, Southworth PM, et al. Health of people experiencing co-occurring homelessness, imprisonment, substance use, sex work and/or severe mental illness in high-income countries: a systematic review and meta-analysis. J Epidemiol Community Health. 2021;75(10):1010–18. 33893182 10.1136/jech-2020-215975 PMC8458085

[57] Watson DP, Shuman V, Kowalsky J, Golembiewski E, Brown M. Housing first and harm reduction: a rapid review and document analysis of the US and Canadian open-access literature. Harm Reduct J. 2017;14(1):30. 28535804 10.1186/s12954-017-0158-x PMC5442650

[58] Alsuhaibani R, Smith DC, Lowrie R, Aljhani S, Paudyal V. Scope, quality and inclusivity of international clinical guidelines on mental health and substance abuse in relation to dual diagnosis, social and community outcomes: a systematic review. BMC Psychiatry. 2021;21(1):23. 33892659 10.1186/s12888-021-03188-0 PMC8066498

[59] Bendixen A, Sagvaag H, Ostenstad BH, Gronnestad T. Rettigheter og tvang i kommunale hjelpetilbud til personer med alvorlige samtidige rus- og psykiske lidelser: erfaringer med regelverket, i lys av menneskerettigheter. Nord Stud Alcohol Drugs. 2023;40(6):590–605. 10.1177/14550725231156479 PMC10688403 38045005

[60] Keyser DJ, Watkins KE, Vilamovska AM, Pincus HA. Focus on alcohol & drug abuse: improving service delivery for individuals with co-occurring disorders: new perspectives on the quadrant model. Psychiatr Serv. 2008;59(11):1251–53. 18971399 10.1176/ps.2008.59.11.1251 PMC6545908

[61] Helsedirektoratet. Nasjonal faglig retningslinje for utredning, behandling og oppfølging av personer med samtidig ruslidelse og psykisk lidelse – ROP-lidelser. Sammensatte tjenester – samtidig behandling. Oslo: Helsedirektoratet; 2022.

[62] Socialstyrelsen. Nationella riktlinjer för vård och stöd vid missbruk och beroende. Socialstyrelsen; 2019.

[63] Wallace J. The practice of evidence-based psychiatry today. Advances in psychiatric treatment: the Royal College of Psychiatrists. J Retailing Continuing Prof Devel. 2011;17(5):389–95.

[64] Welfare FIfHa. A scoping review on the methodological frameworks for supporting transferability and implementation of practices in health and care systems. Eur Partnersh Transforming Health Care Syst. 2023.

[65] Furst MA, Bagheri N, Salvador-Carulla L. An ecosystems approach to mental health services research. BJPsych Int. 2021;18(1):23–25. 34287396 10.1192/bji.2020.24 PMC8274404

[66] Osborne SP, Powell M, Cui T, Strokosch K. Value creation in the public service ecosystem: an integrative framework. Public Adm Rev. 2022;82(4):634–45.

