# Supplementary material for: Organising housing and service provision for persons with co-occurring substance use and mental health problems: a scoping review in the ROP Municipal
Source: BMC Psychiatry. 2025 Dec 11;26:42. doi: 10.1186/s12888-025-07621-6 (PMC12801499; doi:10.1186/s12888-025-07621-6)
Supplement: Supplementary file 1 — Supplementary Material 1 [file 12888_2025_7621_MOESM1_ESM.pdf]

Sorsa et al 2025: https://doi.org/10.1186/s12888-025-07621-6

Supplementary file 1. Search process notes and example of two searches.

Search process notes

Cinahl Complete (EBSCO)

n = 832 Continental European countries, English and Scandinavian languages, age limit

Medline & PsychINFO (OVID)

Database Field Guide Ovid MEDLINE(R) ALL 1946 to January 31, 2023, Database Field Guide APA PsycInfo

n = 12 Limited to adults & language & MESH terms used n = 8

Scopus

n = 379 Nordic countries and restricted languages & areas of research

Social Services abstracts (PROQUEST)

n = 67, With limit to country, no age limit available

SOCINDEX

n = 3 After limit to country

Web of Science

n = 168 Nordic countries (8 with "severe mental") + search for reference lists

TS= 168 WITH NORDIC COUNTRIES ONLY, language English + Nordic, no year limit, age limits not in database

Example of two searches

CINAHL 15.2.2023

Title, Abstract, Subject heading n = 832

With age, country *European continental* and language limit

+ norway or sweden or denmark or finland or iceland or Scandinavia did not work

Subject headings: MM Exact Major Subject heading

Diagnosis, Dual (Psychiatry) OR Psychotic Disorders OR Substance Use Disorders

AND

Housing OR Home Environment OR Residential Facilities OR Housing Instability

(Home OR resident OR housing OR apartment OR flat OR shelter* OR accommodation) AND (dual diagn* OR dually diagnos* OR co-morb* OR comorb* OR co-occur* OR cooccur* OR concurr* OR concomit* OR co-exist* OR coexist* OR double OR triple diag* OR multimorb* OR complex* OR wicked problem OR incarceration OR prison* OR jail OR violen* OR "mental disord*" OR SMI OR "serious mental ill" OR "serious mental diagnos" OR schizophreni* OR psych* OR substance* OR addiction) AND (organization* OR coordinat* OR best practice* OR guideline* OR health serv* OR social serv* OR service model OR harm reduction OR integrated service* OR continu* OR strength* OR motivational* OR comprehensive* OR assertive outreach OR support* OR functiona* OR cultural sensitive* OR competenc* OR integrated* OR community OR complex* OR welfare serv* OR multisect* OR multidisciplin* OR transdisciplin* OR network* OR positive mental health OR inclusion OR flexibl* OR active OR capab* OR consumer participation OR dialog* OR co-ordinat* OR management OR program OR multidimension* OR private OR public OR system* OR recover* OR self-help* OR third sector OR volunteer OR non-profit)

AND

Subject headings MH Exact:

Diagnosis, Dual (Psychiatry) OR Psychotic Disorders OR Substance Use Disorders

OR

Housing OR Home Environment OR Residential Facilities OR Housing Instability

AND

Subject: Major Heading

Xhome environment

Xhousing

Xmental disorders

Xresidential facilities

Xhome health care

Xhomeless persons

Xcommunity living

Xactivities of daily livin...

Xmental health

Xsubstance use disorders

Xrisk assessment

Xmental health services

Xphysical activity

Xprimary health care

Xpsychological well-being

Xself care

WEB OF SCIENCE 15.2.2023

Nordic countries n = 168 (8 with “severe mental”) + search for reference lists

TS = 168 WITH NORDIC COUNTRIES ONLY, language English + nordic, no year limit

SU=Research Area

Clinical & Life Sciences OR Social Sciences

AND

WC=Web of Science Categories

“Education & Educational Research” OR “Family Studies” OR “Health Care Sciences & Services” OR “Health Policy & Services” OR “Humanities, Multidisciplinary” OR Management OR “Medicine, General & Internal” OR “Multidisciplinary Sciences” OR Nursing OR “Public, Environmental & Occupational Health” OR Rehabilitation OR “Social Issues” OR “Social Sciences, Interdisciplinary” OR “Social Work” OR Sociology OR “Substance Abuse”

SEARCH TERMS

Home OR resident OR housing OR apartment* OR flat OR shelter* OR accommodation

AND

“dual diagn” OR “dually diagnos” OR co-morb* OR comorb* OR co-occur* OR cooccur* OR concurr* OR concomit* OR co-exist* OR coexist* OR “double or triple diag” OR multimorb* OR complex* OR “wicked problem” OR incarceration OR prison* OR jail OR violen* OR assault* OR "anti social" OR aggress* OR deviant OR attack OR crimin*OR “mental disord*” OR SMI OR “serious mental ill” OR “serious mental diagnos” OR schizophreni* OR psych* OR substance* OR addiction

AND

organization* OR coordinat* OR “best practice” OR “guideline” OR “health serv” OR “social serv” OR “service model” OR “harm reduction” OR "integrated service" OR continu* OR strength OR motivational OR comprehensive* OR “assertive outreach” OR support OR functiona* OR “cultural sensitiv” OR competenc* OR integrated* OR community OR complex* OR “welfare serv” OR multisect* OR multidisciplin* OR transdisciplin* OR network* OR “positive mental health” OR inclusion OR flexibl* OR active OR capab* OR “consumer participation” OR dialog* OR co-ordinat* OR management OR program* OR quality OR multidimension* OR private OR public OR system* OR recover* OR self-help* OR third sector OR volunteer* OR non-profit

TS= 2005 WESTERN COUNTRIES, language English + nordic

TS= 1321 WESTERN COUNTRIES, language English +Nordic, year limit 2013-
