# Supplementary material for: Organising housing and service provision for persons with co-occurring substance use and mental health problems: a scoping review in the ROP Municipal
Source: BMC Psychiatry. 2025 Dec 11;26:42. doi: 10.1186/s12888-025-07621-6 (PMC12801499; doi:10.1186/s12888-025-07621-6)
Supplement: Supplementary file 2 — Supplementary Material 2 [file 12888_2025_7621_MOESM2_ESM.pdf]

SUPPLEMENTARY FILE 2

ROP Municipal, the initial coding scheme 2024
Sorsa et al 2025:
https://doi.org/10.1186/s12888-025-07621-6

Housing service organization, coordination and service provision for persons with COP

NAME OF ARTICLE

How is COP defined? (open answer)

Which organizational level is studied? Responsibilities (in strategic guidelines)

Building trust between care provider and tenant mentioned? (Yes/No)

    Responsibilities discussed? (open answer)

Internal organization in housing facilities mentioned? (Yes/No)

    Responsibilities discussed? (open answer)

Regional/municipality level mentioned? (Yes/No)

    Responsibilities discussed? (open answer)

National level mentioned? (Yes/No)

    Responsibilities discussed? (open answer)

Are municipal/regional decisions mentioned? (Yes/No) **Responsibilities (in strategic guidelines)**

What? (open answer)

Housing

What type of housing is mentioned and studied? (e.g. Housing First, single flats, or Bofelleskap/"Communion housing")? (Open question)

National housing guidelines, argumentation & choices mentioned? (Yes/No)

    What (open answer)?

Other issues (e.g. architecture or aesthetics mentioned? (Yes/No)

    What (open answer)?

What approaches and orientations have been used? (e.g. resilience and recovery, positive mental health) in strategic guidelines? (Give examples)

Recovery orientation as a non-diagnostic approach? (Yes/No)

Harm reduction? (Yes/No)

Health promotion and physical well-being? (Yes/No)

SUPPLEMENTARY FILE 2

ROP Municipal, the initial coding scheme 2024
Sorsa et al 2025:
https://doi.org/10.1186/s12888-025-07621-6

Floating, being flexible, or rule bending? (Yes/No)

Client-centeredness, individuality, and gender-specific choices? (Yes/No)

Networking, collaborative processes? (Yes/No)

Connectedness to community (felleskap) and social community support? (Yes/No)

Participation, user involvement, and citizenship (medborgerskap)? (Yes/No)

Meaningful activities or work, and meaningfulness in everyday life? (Yes/No)

Staff support for recovery processes (i.e. connectedness, hope, identity, meaning and empowerment)? (Yes/No)

Joint reflection, respect, and acceptance? (Yes/No)

Peer support? (Yes/No)

Involvement of family members (e.g. children) and/or significant others? (Yes/No)

Importance of building relationships

System of care providers? (Yes/No)

Relationship between workers and inhabitants? (Yes/No)

Therapeutic relationships? (Yes/No)

Organization as a whole (in strategic guidelines)

Knowledge based practice / best available knowledge /unclear (Open answer)

Multidisciplinary approach and integration of services? (Yes/No)

What?

Accessibility and availability (availability of flats)? (Yes/No)

What?

Individual care planning (e.g. of practical care in everyday life in household)? (Yes/No)

Individual plan (e.g. for overall future goals and plan -in Norway by law) (Yes/No)

Resources?

What?

Staff expertise in housing? (Yes/No)

Which professionals (e.g. health and social professionals)? (open answer)

User experience (brukarerfaring), as staff? (Yes/No)

Skills development in systems of care (in strategic guidelines)? (Yes/No)

SUPPLEMENTARY FILE 2

ROP Municipal, the initial coding scheme 2024
Sorsa et al 2025:
https://doi.org/10.1186/s12888-025-07621-6

Economics mentioned? (Yes/No)

Continuous evaluation? (Yes/No)

Organisation, practical work, and type of evaluation?

Follow up on the well-being of tenants

Housing stability? (Yes/No)

Satisfaction? (Yes/No)

Substance use? (Yes/No)

Contact with social services? (Yes/No)

Reason for using service? (open answer)

Safety in home? (Yes/No)

Quality of care? (open answer)

Other? (open answer)

Operational rules and principles in housing

Limits? (open answer)

Safety and security? (open answer)

Other? (open answer)

Values, ideals and treatment philosophy (in strategic guidelines)

Ethical argumentation? What? (open answer)

Independence, autonomy, or citizenship (in strategic guidelines)? (Yes/No)

Rights of users, human rights, dignity (i.e. in strategic guidelines), hope, perseverance (i.e. “Always a new chance and support”)? (open answer)

What kind of problems exist, and need to be solved on organisational level? (open answer)

Best-practice guidelines or recommendations? (Yes/No)

What?

In which areas?
